# Bone Density as a Marker of Response to Radiotherapy in Bone Metastatic Lesions: A Review of the Published Data

**DOI:** 10.3390/ijms17091391

**Published:** 2016-08-24

**Authors:** Vassilis Kouloulias, Zoi Liakouli, Anna Zygogianni, Kyriaki Mystakidou, John R. Kouvaris

**Affiliations:** 1 2nd Department Radiology, Radiotherapy Unit, Medical School, National Kapodistrian University of Athens, ATTIKON University Hospital, 12462 Athens, Greece; 2 1st Department Radiology, Radiotherapy Unit, Medical School, National Kapodistrian University of Athens, Aretaieion University Hospital, 11528 Athens, Greece; zliakouli@yahoo.gr (Z.L.); annazygo1@yahoo.gr (A.Z.); mistakidou@yahoo.com (M.K.); johnkouvaris@gmail.com (J.R.K.)

**Keywords:** bone metastasis, radiotherapy, bone density

## Abstract

Metastases to the bone are presenting in a great percentage of patients with cancer, causing a variety of symptoms, affecting the quality of life and survival of patients. A multidisciplinary approach from different health providers is required for treatment, including radiation oncologists, medical oncologists and surgeons. The role of radiotherapy in the management of bone metastases has long been established through multiple randomized trials. The estimation of response to the therapy is subjective and is based on the palliation of the symptoms that the patients report. However, a quantification of the tumor burden and response to the treatment with the use of an objective method to measure those parameters is a clinical expectation in oncology. The change in bone density in affected areas (mainly lytic) after local radiotherapy, representing the cellular changes that have occurred, is a promising marker of response to treatment.

## 1. Introduction

Bone metastasis present very commonly in the course of solid tumors, especially in the case of breast, prostate and lung cancer. On 31 December 2008, approximately 280,000 adults were estimated to be living with metastatic bone disease in the US [1]. Malignant disease of the bone has a great impact in the quality of life of cancer patients, as a result of pain, pathological fractures, impaired mobility, hypercalcemia, and neurological complications caused by spinal cord compression [2], and it can also negatively affect the survival of these patients [3,4].

The main goal of treatment is palliation of symptoms and treatment options include surgery for decompression of the spinal cord and stabilization of impending fractures, radiotherapy, radioisotopes and systemic therapy, in terms of chemotherapy, hormonal therapy and the use of biphosphonates [5]. Radiotherapy is an effective treatment for the palliation of pain, as has been demonstrated in multiple randomized trials of different fractionation schedules [6,7,8,9,10,11,12,13,14]. Up to 60% of the patients report some degree of palliation of pain after radiotherapy and 24% report complete response to the treatment, results that are similar in different trials [6]. There is no statistically significant difference in the effectiveness in the reduction of pain between different fractionation schedules of radiotherapy (single versus multiple fractions) [7], with the exception of neuropathic pain, where single fraction radiotherapy was not shown to be as effective as multi-fraction therapy, in a randomized trial by Roos et al. [8].

Palliation of pain, however, is a subjective way of measuring the response to therapy, and for that reason bone metastases have been considered “non-measurable”, in contrast with lesions in other organs, such as lung and liver, and the identification of a marker for assessing response is of great importance. The aim of this review was to evaluate the published data regarding the use of bone density as a reproducible and objective way to measure treatment response to radiotherapy in osteolytic bone metastases.

## 2. Search Procedure and Published Data

Pubmed, EMBASE and the Cochrane Library were searched for articles including the keywords bone metastases, radiotherapy and bone density from 2000 up today. Abstracts in supplements and oral presentations were not included. Four articles were identified that quantitatively measured the change in bone density after radiotherapy in patients with bone metastasis. The published data are shown in Table 1.

Kouloulias and colleagues reported the results of a study that included 42 patients and evaluated the efficacy of the combination of radiotherapy with the biphosphonate pamidronate compared with radiotherapy alone. Primary endpoints were the mean value and energy of gray level histogram in plain radiographs (MVGLH and EGLH) and the relative electron density (RED) of bone lesions measured in CT scans [15].

Vassiliou et al., in a total of 52 patients, studied the effect that radiotherapy had in combination with the use of biphosphonates in a variety of parameters, including pain relief, quality of life, Karnofsky Performance Status and bone density. Bone lesions were divided in lytic, sclerotic and mixed [16]. In another study by Vassiliou et al., 45 patients received the combination of local radiotherapy in affected areas with ibandronate, and opioid use, mean performance status and change in bone density were reported [17].

Foerster et al., in a retrospective study, examined the change in bone density in 152 lytic bone lesions in 115 patients from breast cancer, measured in CT scans [18]. A bone density measurement was also performed in non-affected, irradiated, neighboring bones to examine the change in bone density that could be attributed to the systemic therapy.

Rief et al., in a randomized controlled trial which included 60 patients, used bone density measured in CT images to evaluate the effect that resistance training had in addition to radiotherapy in bone lesions. The rate of pathological fractures was the secondary end point and bone density measurements in unaffected vertebral bodies were performed to detect changes from systemic treatment [19].

## 3. Discussion

Bone is the most common organ affected by metastases and approximately 70% of patients with breast cancer or prostate cancer will develop bone metastases in the course of their disease [2], which is of particular clinical importance, given the prevalence of these two diseases. Radiotherapy is an easy, not-time-consuming, cost-effective [20] treatment for metastatic bone disease, with very good results in the palliation of pain. In a meta-analysis of randomized trials of palliative radiotherapy for bone metastases, up to 70% of the patients had some degree of pain relief [7].

There are different fractionation schedules of radiotherapy, from 8 Gy in one fraction to 30 Gy in 10 fractions, which are used to treat bone metastasis. Multiple randomized trials have compared the effectiveness of these different treatment schedules, showing that there is no statistically significant difference between single- and multi-fraction radiotherapy in terms of palliation of pain [9,10,11,12,13,14]. A systematic review of the randomized trials, though, revealed that patients that were randomized to the single arm group were 2.6 times more likely to require retreatment than those who received multiple fractions [6].

Using pain relief as a measure of response to treatment is subjective and in the case of metastatic bone disease the need for an objective, reproducible marker as in other solid organs is evident. The RECIST criteria that are used for the evaluation of treatment outcome in solid tumors are based mainly on Computerized Tomography (CT) imaging and offer specific guidelines for the quantitative measurement of tumor response [21], offering the clinicians an important tool on the pathway of decision-making for the patient’s management. Bone lesions are considered “non-measurable” according to the RECIST criteria, especially sclerotic ones. Therefore, the use of image processing to assess the effect that radiotherapy has on affected bones seems to be the question that needs to be answered. Plain imaging, by means of conventional X-rays, was used by Kouloulias et al. for the evaluation of recalcification of osteolytic bone metastasis in patients with breast cancer that received the combination of radiotherapy with disodium pamidronate (DP) [22,23]. Plain radiographs were taken at baseline and two weeks after each i.v. infusion of DP, using the same settings for the exposure. The quantitative assessment of bone change was based on measuring the first-order statistics of the mean value and energy of gray-level histograms (MVGLH-μ and EGLH-e, respectively) in the osteolytic region. The quantitative assessment of bone loss was obtained using the mean value and energy in terms of the first-order statistics, as defined below.

If n(I) is the number of pixels whose intensity is I and N is the total number of pixels in the region of interest, then the occurrence probability of intensity I is:

$$P\left(I\right)=\frac{n\left(I\right)}{N}$$

At eight-bit gray-level quantization, the resulting distribution takes the form of a first-order histogram with 256 bins, where each bin is one of the integer sample values *I* = 0, …, 255.

Then the mean value of the gray-level histogram (MVGLH) is assessed as:

$$\mu =\sum_{I=0}^{255}I(PI)$$

estimating the value around which central clustering occurs.

Next, the energy of the gray-level histogram (EGLH) is assessed by:

$$\text{energy}=\overset{255}{\underset{I=0}{\sum }}P\left(I\right)$$

**Figure 1:**
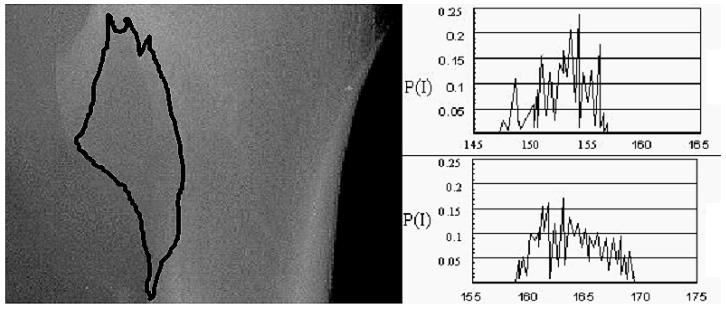
First-order statistics as an image processing technique for the evaluation of recalcification of bone lytic metastasis at the femur after radiotherapy. Mean value and energy (MVGLH, EGLH) at baseline (EGLH = 0.19, MVGLH = 155.1) and six months after initiation of multimodality treatment (EGLH = 0.17, MVGLH = 173.3). *P*(*I*) refers to the probability of intensity (*I*).

As mineral is lost from the lytic metastases, the distribution of pixel intensities is shifted and becomes more concentrated at the lower gray levels. As a result, the mean value decreases and the energy increases in the osteolytic regions. After the evaluation of the images by expert radiologists, significant changes were detected. In detail, there was an 11.08% (95% CI 10.21, 11.93) mean reduction of energy in the gray-level histogram and an 11.63% (95% CI 10.96, 12.29) increase in the mean value of the gray-level histogram, showing a radiological improvement (Figure 1). However, there are some limitations in the use of plain radiographs and MVGLH and EGLH, especially in the case of the thorax and the abdomen where there are superimposed movable tissues. The deviation in MVGLH was studied after sequential radiographs were taken using the same settings and it was found to be higher in areas of the thorax (21.2%) and the abdomen (42.4%), while the deviation in weight-bearing bones was a maximum of 2.9% [24]. The use of an image-processing method that could offer quality assurance and reproducibility, by means of CT images that are used for solid organs, seems appealing.

In this review, we examined also the published data for the use of bone density on CT, a marker that reflects the changes that occur in bones on a cellular and morphological basis, as a way to measure the response to radiotherapy quantitatively. Kouloulias et al., in the study of the combination of radiotherapy and the biphosphonate pamidronate, used the corrections of bone relative electron density *p_e_*, reported by Thomas [25], as it appears in the following formula:

$$Pe=\frac{HU}{1950}+1.0Ps$$

where HU stands for Hounsfield Units, for their quantitative assessments. By comparing the baseline with measurements at six months, RED values of bone lesions were significantly changed for all patients in terms of 1.20 ± 0.30 at baseline to 1.33 ± 0.20 at six months. Measurements in the group of patients that received the combination of radiotherapy and pamidronate were significantly superior to those that received radiotherapy alone. The same results were reported also in the other evaluated parameters, such as BPS, AIS and ECOG performance status, for the combination of radiotherapy with pamindronate, to show superiority over radiotherapy alone. Although the trial was non-randomized, it showed that image-processing in plain radiology is a simple and reliable tool for monitoring changes in bone formation [15].

Vassiliou et al., in their study of measuring the change in mean bone density in three different bone lesions (lytic, sclerotic, mixed), reported that there was a statistically significant increase in mean bone density in all groups, almost by three-fold in lytic lesions at a period of 10 months, a two-fold increase in the same period for the mixed lesions and an increase in the mean bone density of 138 HU for the sclerotic lesions. A statistically significant difference was also observed in the other parameters in all the groups, including pain relief, opioid consumption, quality of life (QOL) and Karnofsky Performance Status. However, there were some differences between the three groups. Patients in the lytic group had the highest mean score pain at baseline, the highest percentage of opioid consumption, and the lowest mean score for physical functioning and performance status. That group, though, also showed significant pain relief by means of 64.3% reporting a complete response at 10 months, when in the mixed group 75% reported a complete response in the same period and in the sclerotic group 83.3% of patients experienced a complete response at 10 months. Similar were the differences in opioid consumption, QOL and Karnofsky Performance Status (PS). Despite the small number of patients in this study, the mean bone density measured by CT was a practical and efficient way of monitoring the response to treatment [16]. In another study by Vassiliou, patients received ibandronate in combination with radiotherapy and changes in mean bone density were reported at three, six and 10 months. An evaluation of the mean physical functioning, using the EORTC QLQ-C30 questionnaire (EORTC Data Center, Brussels, Belgium), Karnofsky Performance Status and opioid use, was also performed. Mean bone density increased significantly at all time points and after 10 months it was 73.2% higher than baseline. A correlation was determined between clinical and radiological parameters and a positive correlation was found between the mean bone density and mean physical functioning at three, six and 10 months (*R*_S_ = 0.35, 0.58 and 0.66, respectively). Bone density in CT appeared to be a precise and quantifiable method of measuring changes in bone lesions [17]. 

In a recent retrospective analysis by Foerster et al., bone density was assessed in HU in 152 lytic lesions and in neighboring unaffected areas to examine the effect of systemic therapy, three and six months after radiotherapy. Bone density increased significantly in the affected areas during follow-up, by a mean of 145.8 HU ± SD (Standard Deviation) 139.4 (*p* < 0.0001) at three months and by a mean of 238.0 HU ± SD 149.2 (*p* < 0/0001) at six months. The mean bone density of the irradiated unaffected neighboring areas showed a slight decrease of −7.3 HU ± SD 60.4 HU at three months and at six months there was practically no change in bone density. Patients that did not receive biphosphonates had an increase in bone density of 76.03 HU ± SD 86.6 (*p* = 0.069) [18], data that are in accordance with the results of the study by Kouloulias [15], showing that biphosphonates have radiosensitizing effects. A univariate analysis of other possible prognostic factors, such as chemotherapy and hormonal therapy, hormone receptor status, Karnofski Performance Status (KPS), the overall applied radiotherapy dose and the use of a surgical corset, did not appear to correlate with the change of bone density after radiotherapy.

The German Bone Research Group conducted a phase III randomized trial to examine the effect of resistance training in addition to the standard of care treatment, radiotherapy, in spinal metastases. CT-based bone density was used as a tool for evaluation of treatment response and measures were performed in non-affected neighboring vertebral bodies to rule out bias from the use of systemic therapy. Both lytic and sclerotic lesions were included. Bone density increased significantly after six months from radiotherapy, both in the control group and the resistance training group. However, there was a statistically significant increase in bone density between the two groups, underlying the impact of resistance training. Osteoblastic lesions showed no significant increase in bone density between the two groups and there was no significant difference in the rate of pathological fractures, as a result of resistance training. The measurements from the uninvolved bones showed no significant difference between or within the two groups, results that were in accordance with the study from Foerster et al., showing that systemic treatment had no crucial impact on bone density.

Authors conclude that bone density measured in HU appears to be a reliable and reproducible method for the evaluation of response to treatment. However, there are some limitations in the use of CT-based bone density as a measure tool that need to be taken into consideration, as a result of the subjective manual measuring. 

## 4. Conclusions

Bone density appears to be a reproducible, reliable and quantifiable marker for measuring the response to radiotherapy in metastatic bone disease in a quantitative way. Furthermore, it reflects the increase of stability of the irradiated bone and is positively correlated with clinical improvement. Therefore, the results of this tool can give evidence for improvement in mobility and quality of life in the daily routine. The use of such a marker to compare the effectiveness of different fractionation schedules of radiotherapy objectively could be tested in a randomized trial.

## Figures and Tables

**Table 1:** Relevant studies with parameters related to bone mineral density for radiation therapy to bone metastases.

Study	Number of Patients	Evaluated Parameters
Kouloulias et al. [15]	42	MVGLH, EGLH, RED, BPS, AIS, ECOG status, UHPC
Vassiliou et al. [16]	52	Pain relief, opiod use, QOL, Karnofky Performance Status, bone density
Vassiliou et al. [17]	45	Pain relief, opioid use, physical phunctioning, QOL, bone density, MRI
Foerster et al. [18]	115	Bone density
Rief et al. [19]	60	Bone density, pathological fractures

MVGLH: mean value gray level histogram; EGLH: energy gray value histogram; RED: relative electron density, BPS: bone pain score; AIS: analgesic intake scale; ECOG: Eastern Cooperative Oncology Group; UHPC: urine hydroxyproline-creatine ratio; QOL: quality of life; MRI: Magnetic Resonance Image.

## Abbreviations

AIS	Analgesic intake scale
BPS	Bone pain score
CI	Confidence Interval
CT	Computed tomography
DP	Pamidronate disodium
ECOG	Eastern Cooperative Oncology Group
EGLH	Energy gray value histogram
EORTC QLQ-C30	European Organization for Research and Treatment of Cancer Quality of Life Questionnaire C-30
i.v	Intravenous
HU	Hounsfield Units
KPS	Karnofski Performance Status
MRI	Magnetic Resonance Image
MVGLH	Mean value gray level histogram
QOL	Quality of life
RED	Relative electron density
SD	Standard Deviation
UHPC	Urine hydroxyproline-creatine ratio
